# Synergistic Application of Platelet-Rich Fibrin and 1% Alendronate in Periodontal Bone Regeneration: A Meta-Analysis

**DOI:** 10.1155/2019/9148183

**Published:** 2019-08-18

**Authors:** Feifei Li, Peipei Jiang, Jinhai Pan, Chengcheng Liu, Liwei Zheng

**Affiliations:** ^1^State Key Laboratory of Oral Diseases, National Clinical Research Center for Oral Diseases, West China Hospital of Stomatology, Sichuan University, Chengdu, China; ^2^West China School of Stomatology, Sichuan University, Chengdu, China

## Abstract

Periodontal bone regeneration relies on coupled and cooperative bone formation and resorption. Accordingly a novel strategy on concurrent use of platelet-rich fibrin (PRF) (anabolic agent) and 1% alendronate (ALN) (anticatabolic agent) was proposed recently in regenerative periodontal treatment. It was supposed to enhance bone formation and reduce bone resorption simultaneously. However, there is a lack of evidence-based studies to answer whether this concurrent application was superior to single application until now. Besides, concerns on ALN lead to some reservation on this synergistic way. ALN may impair new bone formation and necrotize jaws. Thus, in order to compare the clinical efficacy between PRF plus 1%ALN and PRF alone on periodontal bone regeneration, we performed present systematic review and meta-analysis. Because it is the prerequisite for measuring the combined efficacy of PRF plus 1%ALN, firstly we evaluated the effectiveness of 1%ALN. Our data indicated that adjunctive 1%ALN was effective in promoting periodontal bone repair. Further, PRF plus 1%ALN showed a greater capacity for periodontal regeneration than PRF alone with statistical significance. The findings of this study revealed the promising prospects on synergistic application of bone anabolic agents (PRF) and antiresorption medications (1%ALN) in regenerative periodontal treatment.

## 1. Introduction

Regenerative medicine in skeletal system always attracts clinicians and researchers [1, 2]. Fortunately, recent decades witness the conceptual progress of bone biology, which in turn contributes to new therapeutic strategies managing bone loss diseases [3, 4]. Bone formation and resorption are viewed as two major events during bone regeneration [5]. Based on this conception, bone anabolic treatments and antiresorption ways were introduced individually [1, 6]. Certain bone anabolic agents, such as PTH, recombinant human growth factors, and platelet-rich concentrates, as well as the major antiresorption medications, namely, bisphosphonates (BPs), have already been showed to be effective in regenerating periodontal bone [1, 7]. Moreover, since bone formation and resorption are coupled and coordinated with each other, concurrent application of anabolic and antiresorption agents are introduced in regenerative periodontology very recently [8–10]. However, there is a lack of evidence-based studies to answer whether this synergistic way was superior to single application until now. In order to answer this question, we did present systematic review and meta-analysis.

Synergistic application of platelet-rich fibrin (PRF) and 1% alendronate (ALN) is one promising combined strategy in regenerative periodontal treatment [10–12]. In 2001 Choukroun et al. first developed PRF [13]. It is a complicated assembly of white blood cells, platelets, glycanic chains, and structural glycoproteins within a fibrin scaffold. The fibrin network further serves as a reservoir of varies growth factors which could be continuously released for 10 to 14 days [14]. These components are essential players in tissue would healing and together are able to facilitate angiogenesis, cell proliferation and differentiation, which leads to new bone and tissue regeneration subsequently [14]. Thus PRF is characterized as a bone anabolic agent [7]. Conversely, ALN is a typical BP which targets at osteoclastogenesis. It mighty impairs osteoclast differentiation and activities to reduce bone resorption [15]. Topical application of 1%ALN has already been reported to be potently beneficial in reconstructing periodontal bone [15, 16].

Thus the original motivation for combining PRF with 1%ALN in regenerative periodontal treatment is to promote bone formation and inhibit bone resorption at the same time. However this combined strategy is confronted with an inevitable question that dose ALN really benefit the efficacy of PRF or conversely impair it? As ALN targets osteoclasts survival and activation it may break resorption-coupled new bone formation [17]. One of the heavily reported side-effects of ALN is new bone formation impairment owing to excessive resorption-inhibition [17]. Bone healing needs tissue remodeling which includes both soft callus and hard callus remodeling, so it requires the corporation of bone formation and bone resorption [9]. Moreover many sorts of BPs, including ALN, were reported to be potential risk factors of medication-associated osteonecrosis of jaw (MAONJ) though relative mechanisms remain largely unclear [18]. These two concerns make it uncertain whether uniting 1%ALN and PRF is a worthy and beneficial strategy.

To answer this question, we firstly evaluated the effectiveness of 1%ALN on periodontal bone repair, because it is the prerequisite for further evaluation of concurrent application of PRF plus 1%ALN. Although there has been two systematic review addressing this topic, we noticed several new randomized controlled clinical trials (RCTs) emerged after these publications [15, 16]. Hence necessary updating is called for. On this basis, present meta-analysis for the first time addressed the question that whether synergistic application of anabolic (PRF) plus antiresorption (1%ALN) agents was superior to single application of PRF. According to our meta-analysis, 1%ALN alone was effective on elevating periodontal bone regeneration. Further, PRF plus 1%ALN showed a greater capacity for periodontal regeneration than PRF alone with statistical significance. The findings of this study potentially revealed the promising prospects on synergistic application of PRF and 1%ALN in regenerative periodontal treatment.

## 2. Methods

This study was performed in strict accordance with the Cochrane Handbook for Systematic Reviews of Interventions and Preferred Reporting Items for Systematic Reviews and Meta-Analyses (PRISMA) [19, 20]. The research protocol was modified from two previous studies [21, 22].

### 2.1. Inclusion Criteria

#### 2.1.1. Types of Studies

Only randomized controlled clinical trials (RCTs), including parallel RCTs and split mouth RCTs, were considered for inclusion. Controlled clinical trials (CCTs), quasi-RCTs, cohort studies, case reports, and other studies that fall outside of the category RCTs were excluded. Each RCT included needs to be approved and guided by a reliable ethics committee.

#### 2.1.2. Types of Participants

Patients with periodontitis suffering from periodontal bone loss including vertical inter-proximal bone defect or class II furcation defect were included. Patients with a history of systemic antiosteoporosis treatments (such as BPs and PTH) or receiving antibiotics or any periodontal therapy in the preceding 6 months were excluded.

#### 2.1.3. Types of Interventions

To compare PRF plus 1%ALN and PRF alone, subjects are allocated to experimental and control group based on having open flap debridement (OFD) with PRF plus 1%ALN or OFD with PRF alone. To evaluate the effectiveness of 1%ALN applied locally on periodontal bone repair, patients only receiving conventional periodontal treatments, including scaling and root planning (SRP), OFD or guided tissue regeneration, were regarded as control group, meanwhile those receiving these conventional periodontal treatments plus 1%ALN were regarded as intervention group.

#### 2.1.4. Types of Outcome Measures

Intrabony defect depth (IBD) reduction and IBD reduction% at the end point of follow-up were selected as the primary outcomes, because the targeted outcome of present meta-analysis was periodontal bone repair. IBD was defined as the radiographic distance from a fixed reference point (the crest of the alveolar bone, the adjacent cuspal tip, or cement-enamel junction) to the most apical point of the base of the defect. The secondary outcomes included pocket probing depth (PPD) reduction, vertical clinical attachment level (VCAL) regained and horizontal clinical attachment level (HCAL) regained

#### 2.1.5. Incidence of Other Complications

All reports on adverse effects of 1%ALN or PRF plus 1%ALN in related studies were taken into consideration seriously in this systematic review.

### 2.2. Exclusion Criteria

Published clinical trials were excluded if they did not meet the above criteria.

### 2.3. Search Methods

The search was restricted to articles written in English. A literature search was carried out within the Cochrane Central Register of Controlled Trials (CENTRAL; 2018), PUBMED (1960 to December 2018), MEDLINE (via OVID, 1948 to December 2018), Embase (1984 to December 2018), the China National Knowledge Infrastructure (CNKI; 1979 to December 2018), and the China Biology Medicine disc (CBM; 1978 to December 2018) and Google Scholar. The online databases of Journal of Dental Research, Journal of Dentistry, Journal of Periodontology, Journal of Clinical Periodontology were also searched. References listed in published articles were also checked. In order to find ongoing clinical trials, the World Health Organization International Clinical Trials Registry Platform was searched. The search strategy is combining both MeSH heading words and free key text words. The MeSH heading words included “PRF”, “platelet rich fibrin”, “platelet rich concentrates”, “bisphosphonates”, “alendronate” and “ALN”. We combined these words with free key text words, such as “periodontal”, “periodontology”, “periodontics”, “periodontitis”, “periodontal disease”, “bone” “bone repair”, or “bone regeneration”. Search strategies were finally combined with the Cochrane Highly Sensitive Search Strategy to identify RCTs.

### 2.4. Study Inclusion

Three reviewers (FFL, PPJ, and LWZ) independently screened and measured the titles and abstracts of potential articles according to the preestablished selection criteria. Then full texts were further assessed for all studies that possibly met the inclusion criteria or for cases in which it was difficult to make a final decision because of insufficient information. When disagreements came up, they were resolved by consensus, and an alternative investigator (JHP) acted as an arbiter when no consensus was reached.

### 2.5. Assessment of Risk of Bias

The Cochrane “risk of bias” instrument was used. Bias evaluation was performed by 3 independent reviewers (FFL, PPJ, and LWZ). Disagreements were resolved by discussion until consensus was reached. The risk of bias was classified into three categories:

(a) Low risk of bias if all domains were marked as “low risk”;

(b) Moderate risk of bias if no domain was marked as “high risk” but at least one was coded as “unclear risk”;

(c) High risk of bias if one or more domains were marked as “high risk.”

### 2.6. Data Extraction

The following data were extracted: demographic data, method of randomization, randomization concealment and blinding, measurement outcomes. Two estimators independently extracted data from the included studies (FFL, PPJ, and LWZ) using a custom-designed form.

### 2.7. Statistical Analysis

Statistical analysis was carried out utilizing Review Manager 5.1. Heterogeneity was assessed via the I^2^ statistic (a test for heterogeneity) on the level of *α*=0.10. If there was considerable or substantial heterogeneity (I^2^> 50%), a random-effects model was adopted; otherwise a fixed-effects model was used. The results of treatment effect were presented as median difference (MD) utilizing 95% confidence intervals (CIs). Statistical significance was calculated at *α*=0.05 (2-tailed z tests).

## 3. Results

### 3.1. Search Results

**Figure 1:**
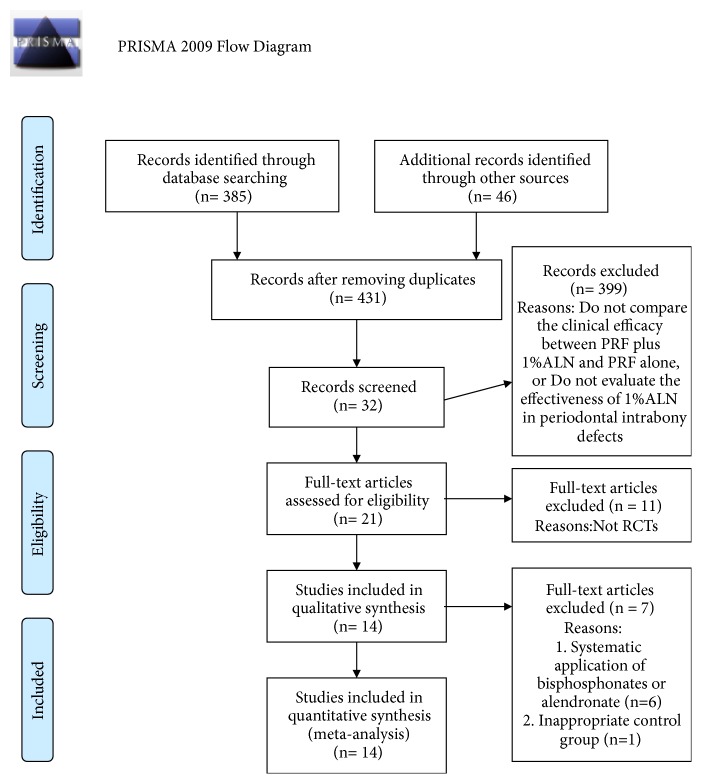
Search results and flow-chart of study selection for meta-analysis. From: Moher D, Liberati A, Tetzlaff J, Altman DG, The PRISMA Group (2009). Preferred Reporting Items for Systematic Reviews and Meta-Analyses: The PRISMA Statement. PLoS Med 6(7): e1000097. doi:10.1371/journal.pmed1000097.

**Figure 2:**
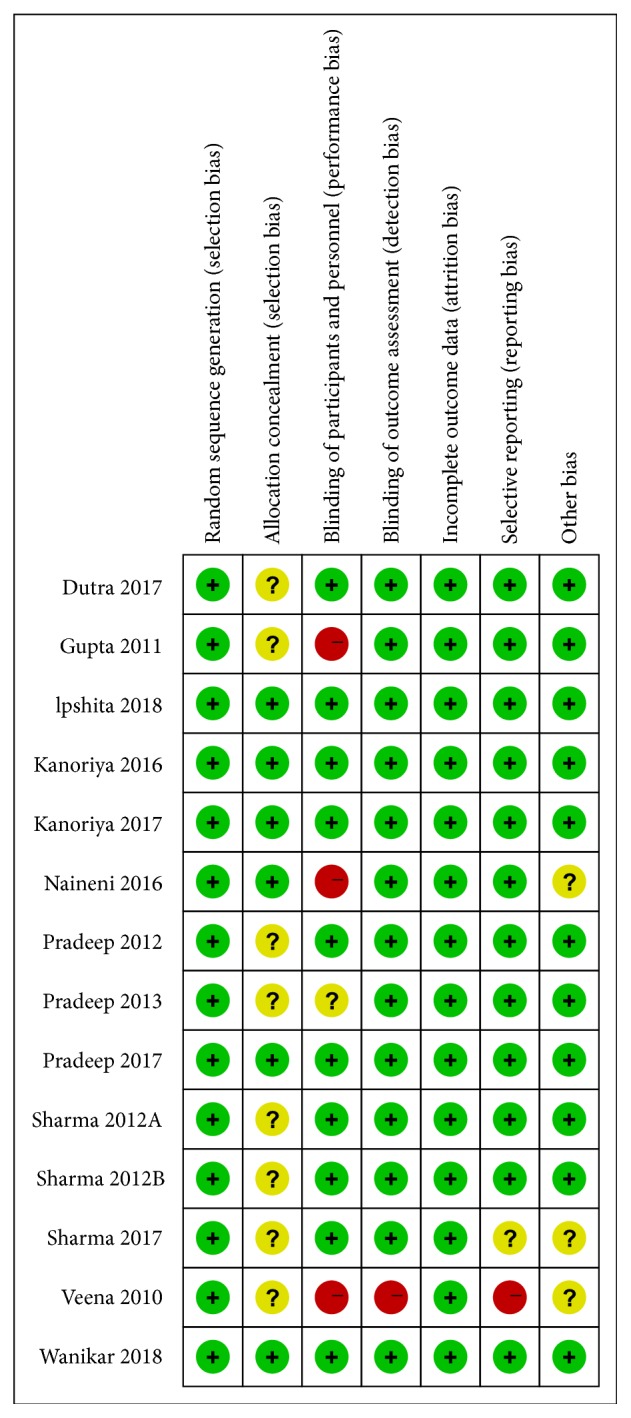
Bias assessment of included studies.

After selection according to our pre-established protocol, 14 RCTs were included for quantitative meta-analysis [10–12, 23–33]. The details of search procedure were presented in study flowchart (Figure 1). Characteristics of included RCTs, including study designs, defect type of enrolled patients, follow-up periods, interventions, controls, and outcomes were detailedly demonstrated in Table 1. Basal condition of patients enrolled in included RCTs was showed in Table S1. The results of bias assessment were showed in Figure 2. Detailed information on bias assessment was provided in Table S2.

### 3.2. Synergistic Application of PRF plus 1%ALN Shows Superior Efficacy on Periodontal Bone Regeneration than PRF Alone

To assess periodontal bone repair, intrabony defect depth (IBD) reduction and IBD reduction% were selected as the primary outcome. Pocket probing depth (PPD) reduction, vertical clinical attachment level (VCAL) regained and horizontal CAL (HCAL) regained were secondary outcomes.

#### 3.2.1. IBD Reduction

**Figure 3:**
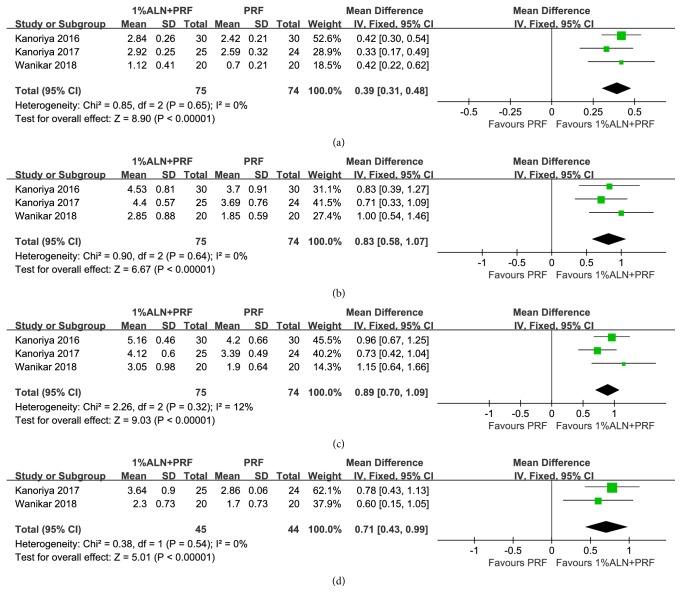
Forest plot of comparison: PRF plus 1%ALN was compared with PRF alone after 6~9 months of follow-up with respect to (a) IBD reduction; (b) PPD reduction; (c) VCAL-regained; (d) HCAL-regained.

According to our data, PRF plus 1%ALN achieved more IBD reduction than that of PRF alone with statistical significance (MD=0.39mm; 95%CI: 0.31, 0.48;* p*<0.00001). Heterogeneity of this meta-analysis was very low (X^2^=0.85, I^2^=0%) (Figure 3(a)). Among included RCTs for this meta-analysis, both Kanoriya 2017 and Wanikar 2018 enrolled patients with degree II furcation defects, while Kanoriya 2016 included patients of three-walled IBD (Table 1). To measure if heterogeneity of enrolled patients would affect the overall effect, we did a subgroup analysis which only enrolled patients with degree II furcation defects. Still data favored PRF+1%ALN over PRF in this patient population (MD=0.37mm; 95%CI: 0.24, 0.49;* p*<0.00001) (Figure S1). The heterogeneity of this meta-analysis was very low (X^2^=0.47, I^2^=0%) (Figure S1). Taken together, synergistic application of PRF plus 1%ALN showed superiority in reducing IBD compared with PRF alone. This effect was consistent in patients suffering from degree II furcation defects or three-walled osseous defects.

#### 3.2.2. PPD Reduction, VCAL-Regained and HCAL-Regained

Meta-analysis on these three parameters also indicated the positive impact of concurrent application when compared with PRF alone. More PPD reduction was showed in PRF+1%ALN group with statistical significance (MD=0.83mm; 95%CI: 0.58, 1.07;* p*<0.00001) (Figure 3(b)). Our data also showed a statistically significant mean difference of both vertical and horizontal CAL-regained between PRF plus 1%ALN group and PRF group in the predicted direction (MD of VCAL regained=0.89mm; 95%CI: 0.70, 1.09;* p*<0.00001) (Figure 3C) (MD of HCAL regained=0.71mm; 95%CI: 0.43, 0.99;* p*<0.00001) (Figure 3(d)). Meta-analysis of these three outcomes showed very low heterogeneity (Figures 3(b), 3(c), and 3(d)).

To sum up, both primary and secondary outcomes proved that synergistic application of PRF plus 1% ALN exhibited significant promotion in regenerating periodontal bone when compared with PRF alone. Considering the high quality of included RCTs and the low heterogeneity of each quantitative outcome, this conclusion was highly reliable (Figures 2 and 3).

### 3.3. Adjunctive 1%ALN is Effective in Regenerating Periodontal Bone

#### 3.3.1. IBD Reduction and IBD Reduction%

**Figure 4:**
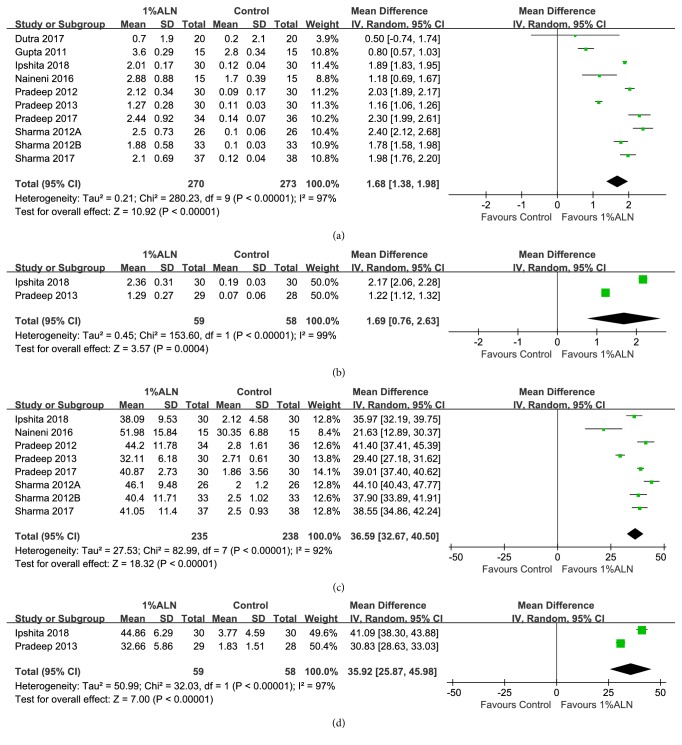
Forest plot of comparison: adjunctive 1%ALN was compared with conventional periodontal treatments alone with respect to (a) IBD reduction after 6 months of follow-up; (b) IBD reduction after 12 months of follow-up; (c) IBD reduction% after 6 months of follow-up; (d) IBD reduction% after 12 months of follow-up.

A total of 10 RCTs reported IBD reduction. They recruited 531 patients in all. Meta-analysis of IBD reduction based on these studies yielded results favorable to 1%ALN. After 6 months of follow-up, IBD reduction in 1%ALN group was 1.68mm higher, constituting a statistically significant difference from the IBD reduction in the control group (MD =1.68mm; 95%CI: 1.38, 1.98;* p*<0.00001) (Figure 4(a)). This effect remained unchanged after 12 months of follow-up (MD =1.69mm; 95%CI: 0.76, 2.63;* p*=0.0004) (Figure 4(b)). In addition, meta-analysis of IBD reduction% also favored adjunctive 1%ALN over conventional treatments with statistical significance. 1%ALN group had 36.59% more IBD reduction% 6 months after surgery (MD =36.59; 95%CI: 32.67, 40.50;* p*<0.00001) (Figure 4(c)). After 12 months of follow-up, patients in 1%ALN group were found to achieve 35.92% higher IBD reduction% than the patients in the conventional treatment group (MD =35.92; 95%CI: 25.87, 45.98;* p*<0.00001) (Figure 4(d)). The heterogeneity of these outcomes was high (Figures 4(a), 4(b), 4(c), and 4(d)).

Among included RCTs, Pradeep 2012 recruited patients with type II diabetes mellitus (DM), and Sharma 2017 enrolled only smoking patients. DM and smoking are widely accepted negative factors for periodontal regeneration. Thus, in order to rule out the potential influence of these two risk factors, we did subgroup analysis which only enrolled systematic healthy patients without smoking habit. Data showed that exclusion of these two studies did not change the overall effect on IBD reduction and IBD reduction% (Figures S2A and S2B). In addition, to measure whether superiority of 1%ALN depended on periodontal defect types, we divided periodontal osseous defects into those with or without degree II furcation defects. Still data favored adjunctive 1%ALN over conventional periodontal treatments alone in both defect types (Figures S2C and S2D).

#### 3.3.2. PPD Reduction, VCAL-Regained and HCAL-Regained

**Figure 5:**
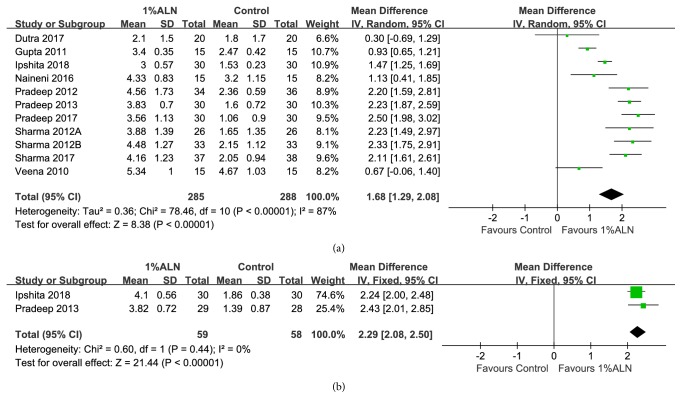
Forest plot of comparison: adjunctive 1%ALN was compared with conventional periodontal treatments alone with respect to (a) PPD reduction after 6 months of follow-up; (b) PPD reduction after 12 months of follow-up.

**Figure 6:**
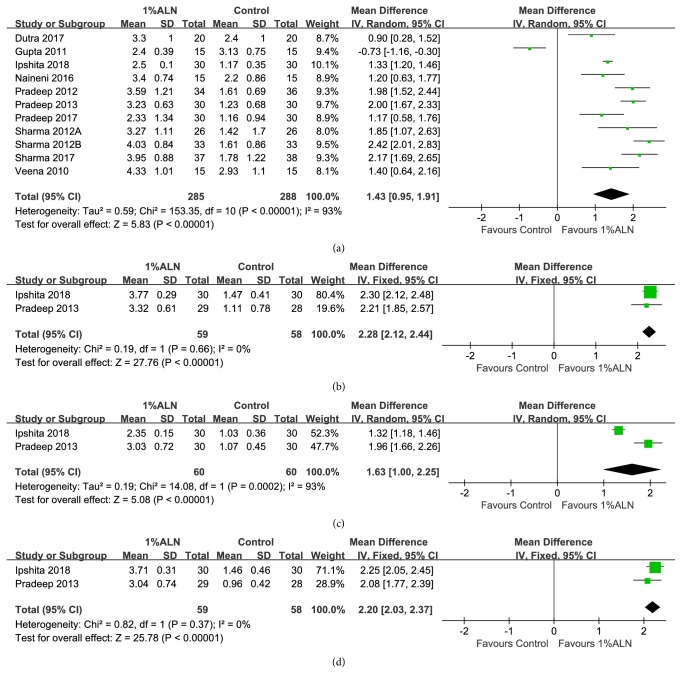
Forest plot of comparison: adjunctive 1%ALN was compared with conventional periodontal treatments alone with respect to (a) VCAL-regained after 6 months of follow-up; (b) VCAL-regained after 12 months of follow-up; (c) HCAL-regained after 6 months of follow-up; (d) HCAL-regained after 12 months of follow-up.

In present meta-analysis, the results for 1%ALN group yielded higher figures for these three secondary outcomes than those found in control group, and the differences were statistically significant (Figures 5 and 6). In exact figures, patients of adjunctive 1%ALN were found to achieve 1.68mm higher PPD reduction than the patients in conventional treatment group after 6 months of follow-up (95%CI: 1.29, 2.08;* p*<0.00001) (Figure 5(a)). They furthermore had 1.43mm more VCAL-regained (95%CI: 0.95, 1.91;* p*<0.00001) and 1.63mm more HCAL-regained than the control groups during this follow-up period (Figures 6(a) and 6(c)). After 12 months of follow-up, 1%ALN group still fared better, with 2.29 higher PPD reduction (95%CI: 2.08, 2.50;* p*<0.00001), 2.28 higher VCAL-regained (95%CI: 2.12, 2.44;* p*<0.00001) and 2.20 higher HCAL-regained (95%CI: 2.03, 2.37;* p*<0.00001) compared to the controls (Figures 5(b), 6(b), and 6(d)).

We also did subgroup analysis which only enrolled systematic healthy patients without smoking habit. Results showed that exclusion of patients with type II DM or smoking habit did not change the overall effect on PPD reduction, VCAL-regained and horizontal CAL-regained (Figures S3 and S4).

## 4. Discussion

Based on updated knowledge of bone biology, it is well known that satisfactory bone regeneration relies on coordinated bone formation and resorption [9]. A new pharmacologic and clinical strategy that aims to simultaneous management of bone formation and resorption, namely concurrent application of PRF and 1%ALN, were proposed recently in order to achieve better periodontal regeneration [10–12]. Previous studies have reported that both PRF and 1%ALN were respectively promising in regenerating periodontal bone [7, 15, 16]. But it remains unclear about the efficacy of the synergistic way. Present study comprehensively reviewed related RCTs and quantitatively measured the efficacy of this synergistic strategy to answer whether PRF plus 1%ALN brings better clinical outcomes than PRF alone. Before this, we analyzed the effectiveness of 1%ALN alone firstly. Because well catching on this issue is the basis of thorough understanding on the role of 1%ALN in the synergistic way.

It should be noticed that concerns of adding PRF with 1%ALN come from potential risks of MANOJ and impairment of new bone formation because of ALN [17, 18]. According to our quantitative results, topical application of 1%ALN is effective in promoting periodontal bone regeneration. No MANOJ cases or other adverse effects were reported in related literature until now. We also found some heterogeneity of patients in our included RCTs. One study enrolled in patients with type II DM and another one recruited smoking patients [25, 29]. All rest RCTs recruited systematic healthy and non-smoking patients. As known that both type II DM and smoking are definite risk factors for periodontitis [34, 35]. Also previous evidences based medicines have clearly elucidated the negative roles of these two risk factors in regenerating periodontal bone [36–38]. Taken these concerns together we did subgroup meta-analysis that ruled out smoking patients and those with type II DM. Results showed that exclusion of these two high risk populations did not change the overall effect of 1%ALN. Taken together, these results revealed the effectiveness of 1%ALN on promoting regeneration of periodontal bone.

Besides, favorable effects of PRF on periodontal tissue healing were also rationales for the synergistic way. A considerable number of RCTs reported application of PRF in regenerative periodontology [39–45]. All these studies demonstrated promising outcomes on periodontal bone repair. The very recent systematic reviews and meta-analysis also proved that PRF was effective in promoting healing of periodontal osseous defects, including intrabony defects and furcation defects [7, 46]. Mechanistically current findings reveal its functions on providing biocompatible scaffolds, continuously releasing cytokines and growth factors, as well as containing profitable cell populations for tissue formation and osteogenesis [14]. In summary, PRF is a promising anabolic agent which can promote bone formation in regenerative periodontology.

Since both 1%ALN and PRF were worthy and beneficial agents, we further conducted the comparison between synergistic PRF plus 1%ALN and PRF alone. Our results of meta-analysis showed that PRF plus 1%ALN achieved more IBD reduction, PPD reduction, vertical and horizontal CAL-regained than that of PRF alone with statistical significance. Our study is the first RCT-based research that supports the superiority of PRF plus 1%ALN over PRF alone in periodontal bone regeneration. But we noticed that all RCTs reporting this combined strategy lacked an important control group which only received 1%ALN [10–12]. Although our meta-analysis showed PRF plus 1%ALN produced much better outcomes on periodontal bone regeneration than PRF alone, it's still necessary to know how much profit are acquired by using PRF plus 1%ALN in comparison to 1%ALN alone. Therefore related RCTs are called for in the future. Because better understandings of this issue will tell us whether we should synergistically use PRF plus 1%ALN or just choose 1%ALN alone.

## 5. Conclusion

In summary, present meta-analysis proved the effectiveness of 1%ALN in periodontal bone regeneration. Further we showed that the synergistic PRF plus 1%ALN brought much better clinical outcomes than PRF alone during periodontal regeneration. These RCTs based evidences potentially endow clinicians with better choices to regenerate periodontal bone. Also they showed the promising prospects of synergistic application of bone anabolic agents and antiresorption medications in regenerative periodontal treatment. It is also clear that more rigorously designed large scale RCTs will be required to confirm or modify the findings that have been suggested by the results of this study.

## Figures and Tables

**Table 1:** Characteristics of included RCTs. F/U: follow-up; PRF: platelet-rich fibrin; ALN: alendronate; OFD: open flap debridement; SRP: scaling and root planing; HA: hydroxyapatite; *β*-TCP: beta-tricalcium phosphate; PPD: pocket probing depth; VCAL: vertical clinical attachment level; HCAL: horizontal clinical attachment level; IBD: Intrabony defect depth.

Study ID	Study Type (Study Design)	Patients (Defect Type)	Control	Intervention	F/U PERIOD (MONTHS)	OUTCOMES
Kanoriya 2016	RCT, parallel	Intrabony defects	RPF+OFD	RPF+1%ALN+OFD	9mons	PPD reduction; VCAL-regained; IBD Depth reduction
Kanoriya 2017	RCT, parallel	degree II furcation defects	RPF+OFD	RPF+1%ALN+OFD	9mons	PPD reduction; HCAL-regained; VCAL-regained; IBD Depth reduction
Wanikar 2018	RCT, split-mouth design	degree II furcation defects	RPF+OFD	RPF+1%ALN+OFD	6mons	PPD reduction; HCAL-regained; VCAL-regained; IBD Depth reduction
Ipshita 2018	RCT, parallel	degree II furcation defects	placebo gel+SRP	1%ALN+SRP	12mons	PPD reduction; HCAL-regained; VCAL-regained; IBD Depth reduction(%)
Dutra 2017	RCT, split-mouth design	Intrabony defects	placebo gel+SRP	1%ALN+placebo gel+SRP	6mons	PPD reduction; VCAL-regained; IBD Depth reduction
Sharma 2017	RCT, parallel	Intrabony defects	placebo gel+SRP	1%ALN+placebo gel+SRP	6mons	PPD reduction; VCAL-regained; IBD Depth reduction(%)
Naineni 2016	RCT, parallel	Intrabony defects	Saline+*β*-TCP+OFD	1%ALN+Saline+*β*-TCP+OFD	6mons	PPD reduction; VCAL-regained; IBD Depth reduction(%)
Pradeep 2017	RCT, parallel	Intrabony defects	placebo gel+SRP	1%ALN+placebo gel+SRP	9mons	PPD reduction; VCAL-regained; IBD Depth reduction(%)
Pradeep 2013	RCT, parallel	degree II furcation defects	placebo gel+SRP	1%ALN+placebo gel+SRP	12mons	PPD reduction; HCAL-regained; VCAL-regained; IBD Depth reduction(%)
Pradeep 2012	RCT, parallel	Intrabony defects	placebo gel+SRP	1%ALN+placebo gel+SRP	6mons	PPD reduction; VCAL-regained; IBD Depth reduction(%)
Sharma 2012 A	RCT, parallel	Intrabony defects	placebo gel+SRP	1%ALN+placebo gel+SRP	6mons	PPD reduction; VCAL-regained; IBD Depth reduction(%)
Sharma 2012 B	RCT, parallel	Intrabony defects	placebo gel+SRP	1%ALN+placebo gel+SRP	6mons	PPD reduction; VCAL-regained; IBD Depth reduction(%)
Gupta 2011	RCT, split-mouth design	Intrabony defects	HA+saline+OFD	1%ALN+saline+HA+OFD	6mons	PPD reduction; VCAL-regained; IBD Depth reduction
Veena 2010	RCT, split-mouth design	Intrabony defects	placebo gel+OFD	1%ALN+placebo gel+OFD	6mons	PPD reduction; VCAL-regained; IBD Depth reduction

## Data Availability

This study is a RCTs-based meta-analysis. All original raw data are provided and accessible in RCTs included in this study (Table 1).

## Supplementary Materials

Supplementary Materials Table S1. Characteristics at baseline of patients enrolled in included RCTs. F/M: female number versus male number; N/A: not available. Table S2. Detailed information on bias assessment. N/A: not available. Figure S1. Forest plot of comparison: PRF plus 1%ALN was compared with PRF alone with respect to IBD reduction in patients with degree II furcation defects after 6-9 months of follow-up. Figure S2. Forest plot of comparison: adjunctive 1%ALN was compared with conventional periodontal treatments alone after 6 months of follow-up with respect to (A) IBD reduction excluding patients with smoking habit or type II diabetes mellitus (DM); (B) IBD reduction% excluding patients with smoking habit or type II DM (C) IBD reduction in patients without degree II furcation defects; (D) IBD reduction in patients with degree II furcation defects. Figure S3. Forest plot of comparison: adjunctive 1%ALN was compared with conventional periodontal treatments alone with respect to PPD reduction in patients without smoking habit or type II DM after 6 months of follow-up. Figure S4. Forest plot of comparison: adjunctive 1%ALN was compared with conventional periodontal treatments alone with respect to VCAL-regained in patients without smoking habit or type II DM after 6 months of follow-up.
