# Supplementary material for: Synergistic Application of Platelet-Rich Fibrin and 1% Alendronate in Periodontal Bone Regeneration: A Meta-Analysis
Source: Biomed Res Int. 2019 Aug 18;2019:9148183. doi: 10.1155/2019/9148183 (PMC6720370; doi:10.1155/2019/9148183)
Supplement: Supplementary Materials — Table S1. Characteristics at baseline of patients enrolled in included RCTs. F/M: female number versus male number; N/A: not available. Table S2. Detailed information on bias assessment. N/A: not available. Figure S1. Forest plot of comparison: PRF plus 1%ALN was compared with PRF alone with respect to IBD reduction in patients with degree II furcation defects after 6-9 months of follow-up. Figure S2. Forest plot of comparison: adjunctive 1%ALN was compared with conventional periodontal treatments alone after 6 months of follow-up with respect to (A) IBD reduction excluding patients with smoking habit or type II diabetes mellitus (DM); (B) IBD reduction% excluding patients with smoking habit or type II DM (C) IBD reduction in patients without degree II furcation defects; (D) IBD reduction in patients with degree II furcation defects. Figure S3. Forest plot of comparison: adjunctive 1%ALN was compared with conventional periodontal treatments alone with respect to PPD reduction in patients without smoking habit or type II DM after 6 months of follow-up. Figure S4. Forest plot of comparison: adjunctive 1%ALN was compared with conventional periodontal treatments alone with respect to VCAL-regained in patients without smoking habit or type II DM after 6 months of follow-up. [file 9148183.f1.pdf]

Synergistic Application of Platelet-Rich Fibrin and 1% Alendronate in Periodontal Bone Regeneration: A Meta-analysis

Feifei Li[1,2,] Peipei Jiang[1,2], Jinhai Pan[1,2], Chengcheng Liu[1,2], Liwei Zheng[1,2#]

1. State Key Laboratory of Oral Diseases, National Clinical Research Center for Oral Diseases, West China Hospital of Stomatology, Sichuan University, Chengdu, China
2. West China School of Stomatology, Sichuan University, Chengdu, China

# Corresponding author: Liwei Zheng
Liwei Zheng: DDS, PhD, State Key Laboratory of Oral Diseases, National Clinical Research Center for Oral Diseases, Department of Pediatric Dentistry, West China Hospital of Stomatology, Sichuan University, No.14, Section 3, South Renmin Road, Chengdu, China, 610041.
Telephone: 862885503469
Fax: 862885503469
mail: liwei.zheng@scu.edu.cn

Supplemental Figure Legends

Table S1. Characteristics at baseline of patients enrolled in included RCTs. F/M: female number versus male number; N/A: not available.

Table S2. Detailed information on bias assessment. N/A: not available.

Fig. S1. Forest plot of comparison: PRF plus 1%ALN was compared with PRF alone with respect to IBD reduction in patients with degree II furcation defects after 6-9 months of follow-up.

Fig. S2. Forest plot of comparison: adjunctive 1%ALN was compared with conventional periodontal treatments alone after 6 months of follow-up with respect to (A) IBD reduction excluding patients with smoking habit or type II diabetes mellitus (DM); (B) IBD reduction% excluding patients with smoking habit or type II DM (C) IBD reduction in patients without degree II furcation defects; (D) IBD reduction in patients with degree II furcation defects.

Fig. S3. Forest plot of comparison: adjunctive 1%ALN was compared with conventional periodontal treatments alone with respect to PPD reduction in patients without smoking habit or type II DM after 6 months of follow-up.

Fig. S4. Forest plot of comparison: adjunctive 1%ALN was compared with conventional periodontal treatments alone with respect to VCAL-regained in patients without smoking habit or type II DM after 6 months of follow-up.

Table S1

Study ID	Patient					
	**Number**	**Age (Year)**	**Gender (F/M)**	**Smoker**	**General Health**	**Defect Type**
Kanoriya 2016	108	30 to 50	55/53	No	Systemically healthy	Intrabony defects
Kanoriya 2017	72	30 to 50	36/36	No	Systemically healthy	degree II furcation defects
Wanikar 2018	20	38 to 56	14/6'	No	Systemically healthy	degree II furcation defects
Ipshita 2018	106	N/A	55/61	No	Systemically healthy	degree II furcation defects
Dutra 2017	20	35 to 60	12/8'	No	Systemically healthy	Intrabony defects
Sharma 2017	46	30 to 50	0/46	Yes	Systemically healthy	Intrabony defects
Naineni 2016	35	30 to 50	17/15	No	Systemically healthy	Intrabony defects
Pradeep 2017	104	30 to 50	51/53	No	Systemically healthy	Intrabony defects
Pradeep 2013	69	30 to 50	32/37	No	Systemically healthy	degree II furcation defects
Pradeep 2012	43	30 to 50	20/23	No	type 2 Diabetes Mellitus	Intrabony defects
Sharma 2012A	20	20 to 35	8/12’	No	Systemically healthy	Intrabony defects
Sharma 2012B	73	30 to 50	34/39	No	Systemically healthy	Intrabony defects
Gupta 2011	15	30 to 50	5/10'	No	Systemically healthy	Intrabony defects
Veena 2010	15	35 to 55	N/A	No	Systemically healthy	Intrabony defects

Table S1. Characteristics at baseline of patients enrolled in included RCTs. F/M: female number versus male number; N/A: not available.

Table S2

ID	Random sequence generation	Allocation concealment	Blinding of participants	Blinding of outcome assessment	Incomplete outcome data	Selective reporting	Other bias
**Kanoriya 2016**	These patients were divided into three groups randomly using a computer	For particular group and treatment allocation, patients were masked	For particular group and treatment allocation, patients were masked	Investigators, operators, and statisticians were masked to the study design	All parameters and enrolled patients were presented in accordance to information in ClinicalTrials.gov NCT02518152	The data report perfectly complies to pre-established protocol	N/A
**Kanoriya 2017**	These patients were randomly divided into three groups (using computer-generated tables).	Patients were masked to group and treatment allocation.	Patients were masked to group and treatment allocation.	Radiograph scanning was done with a scanner § (6,400 dots per inch) for assessment by a person (ARP) who was masked to the participants undergoing surgical therapy.	All parameters and enrolled patients were presented in accordance to information in ClinicalTrials.gov NCT02609061	The data report perfectly complies to pre-established protocol	N/A
**Wanikar 2018**	Coin toss, Lottery, toss of dice, shuffling cards etc(http://ctri.nic.in/Clinicaltrials/showallp.php?mid1=17352&EncHid=&userName=Ishita%20Wanikar)	Centralized (http://ctri.nic.in/Clinicaltrials/showallp.php?mid1=17352&EncHid=&userName=Ishita%20Wanikar)	Participant, Investigator, Outcome Assessor and Date-entry Operator Blinded	Participant, Investigator, Outcome Assessor and Date-entry Operator Blinded	All parameters and enrolled patients were presented in accordance to information in clinical trial registry-India (CTRI/2017/11/010370).	The data report perfectly complies to pre-established protocol	N/A
**Ipshita 2018**	The randomization process was done externally by the statistical unit using a computer-generated random table	investigators were neither involved in the randomization process nor aware of the assigned group in all outcome evaluations.	The present study was a 12-month, longitudinal, parallel-arm, triple-masked study	The present study was a 12-month, longitudinal, parallel-arm, triple-masked study	All parameters and enrolled patients were presented in accordance to information in ClinicalTrials.gov NCT03204097	The data report perfectly complies to pre-established protocol	N/A
**Dutra 2017**	After scaling, the experimental periodontal sites were randomly assigned	No Specific information	Masking: Double (Participant, Outcomes Assessor) (https://clinicaltrials.gov/ct2/show/NCT02470611?t	Masking: Double (Participant, Outcomes Assessor) (https://clinicaltrials.gov/ct2/show/NCT0247061	All parameters and enrolled patients were presented in accordance to information in ClinicalTrials.gov	The data report perfectly complies to pre-established protocol	N/A

			erm=NCT02470611&rank=1)	1?term=NCT02470611&rank=1)	NCT02470611		
**Sharma 2017**	randomly (by computer generated system) assigned to either ALN or placebo group.	No Specific information	Subjects were blinded for allocation into ALN or placebo group.	All pre- and posttreatment clinical parameters were recorded by an examiner (ARP) who was masked to the type of treatment received by the subjects, while another clinician (AS) provided treatment to both groups.	All parameters described in methods were presented in result section	No registrition or pre-established protocol provided	No published protocol as reference
**Naineni 2016**	The patients were screened and eligible patients were randomly assigned by lottery method	Investigator RK concealed allocation	Prior to the initiation of the study, intra-examiner calibration was achieved for the blinded examiners. Preparation	Ethical clearance was obtained from the institutional ethics committee and written informed consent was obtained from all the participants.	All parameters described in methods were presented in result section	The data report perfectly complies to pre-established protocol which was descibed in paper	N/A
**Pradeep 2017**	ARP), patients were randomly (by a computer-generated system) assigned to either the ALN, ATV, or placebo group.	An examiner (ARP) who was masked to the treatment received recorded all pre- and post-treatment clinical parameters, whereas another clinician (VG) provided treatment to both groups.	Patients were masked for allocation	An examiner (ARP) who was masked to the treatment received recorded all pre- and post-treatment clinical parameters, whereas another clinician (VG) provided treatment to both groups.	All parameters and enrolled patients were presented in accordance to information in ClinicalTrials.gov NCT02455869	The data report perfectly complies to pre-established protocol	N/A
**Pradeep 2013**	sites were randomly assigned (by a computer-generated system) to either the ALN (n = 35) or the placebo	No Specific information	No Specific information	All radiographs were reviewed in a single reference center by a masked evaluator (NR).	All parameters and enrolled patients were presented in accordance to information in methods.	The data report perfectly complies to pre-established protocol	N/A

	group (n = 34) (control group) by an evaluator not involved in the study.						
**Pradeep 2012**	participants were randomly (by a computer-generated system) assigned to either the ALN or placebo group	No Specific information	Participants were masked for allocation into the ALN or placebo group.	All pre- and post-treatment clinical parameters were recorded by an examiner (ARP) who was masked to the treatment received	All parameters and enrolled patients were presented in accordance to information in methods.	The data report perfectly complies to pre-established protocol	N/A
**Sharma 2012A**	patients were randomly (by a computer-generated system) assigned to either the ALN group or the placebo group	No Specific information	Patients were masked for allocation into the ALN or placebo groups.	All pretreatment and post-treatment clinical parameters were recorded by an examiner (ARP) who was masked to the type of treatment received by the patients	All parameters and enrolled patients were presented in accordance to information in methods.	The data report perfectly complies to pre-established protocol	N/A
**Sharma 2012B**	patients were randomly (by a computer-generated system) assigned to either the ALN or placebo group	No Specific information	Patients were masked for allocation into the ALN or placebo group.	All pre- and post-treatment clinical parameters were recorded by an examiner (ARP) who was masked to the treatment received	All parameters and enrolled patients were presented in accordance to information in methods.	The data report perfectly complies to pre-established protocol	N/A
**Veena 2010**	The selected sites were randomly asigned as either control or experimental site.	No Specific information	No blinding	No blinding	All parameters and enrolled patients were presented in accordance to information in methods.	No detailed protocol	No detailed and reliable pre-establis hed protocol nor associated information provided

**Gupta 2011**	Sites were selected by simple random sampling technique and assigned as control site (group A) and test site (group B)	No Specific information	After an explanation of the proposed study criteria, including treatments	A controlled, single-blind study was designed, and patients were not masked	All parameters and enrolled patients were presented in accordance to information in methods.	The data report perfectly complies to pre-established protocol	N/A

Table S2. Detailed information on bias assessment. N/A: not available.

Supplemental Figure 1

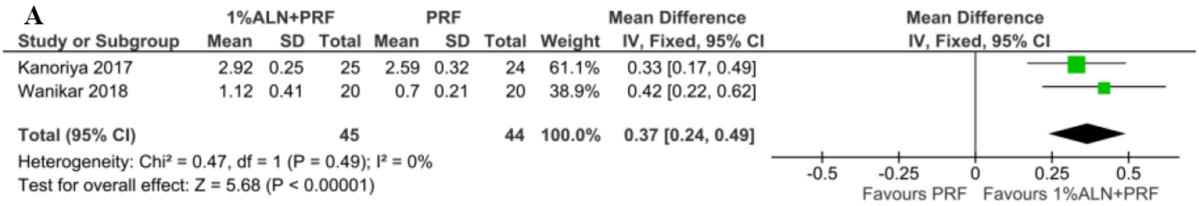

Fig. S1. Forest plot of comparison: PRF plus 1%ALN was compared with PRF alone with respect to IBD reduction in patients with degree II furcation defects after 6-9 months of follow-up.

Supplemental Figure 2

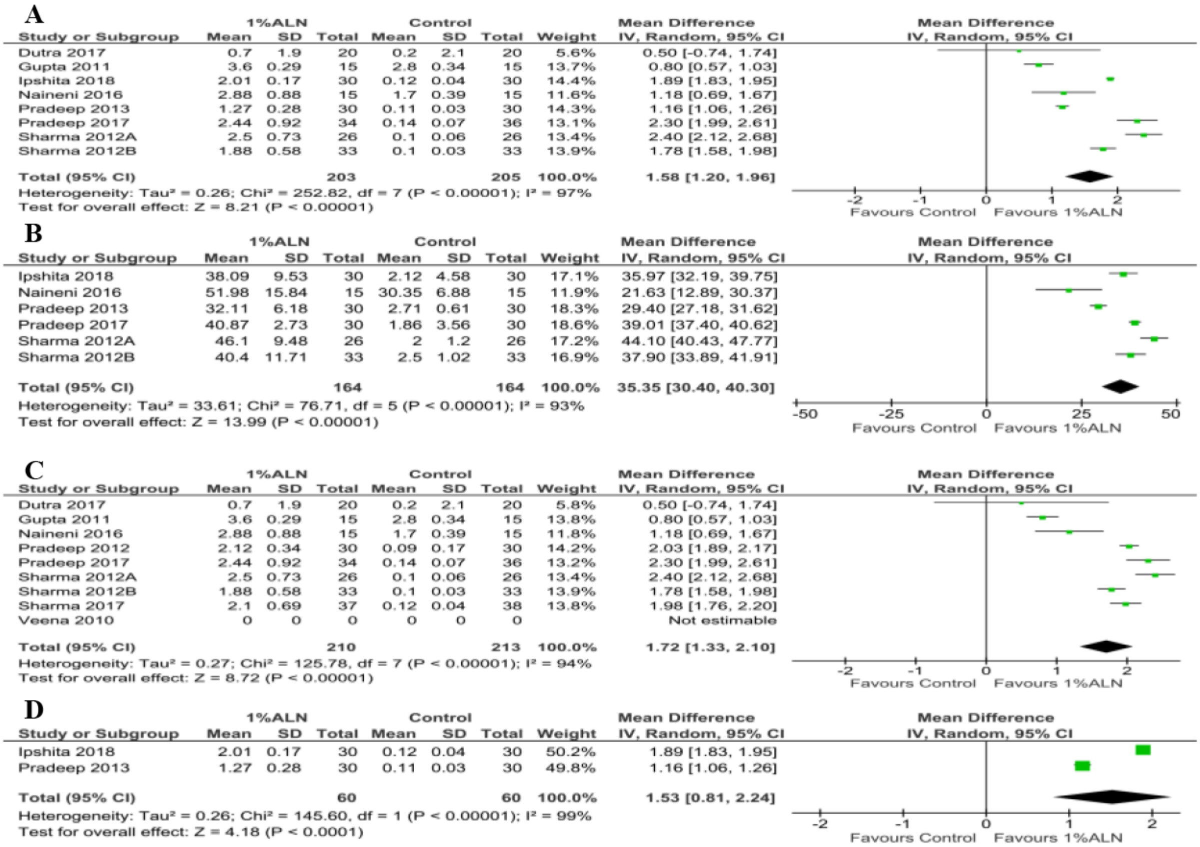

Fig. S2. Forest plot of comparison: adjunctive 1%ALN was compared with conventional periodontal treatments alone after 6 months of follow-up with respect to (A) IBD reduction excluding patients with smoking habit or type II diabetes mellitus (DM); (B) IBD reduction% excluding patients with smoking habit or type II DM (C) IBD reduction in patients without degree II furcation defects; (D) IBD reduction in patients with degree II furcation defects.

Supplemental Figure 3

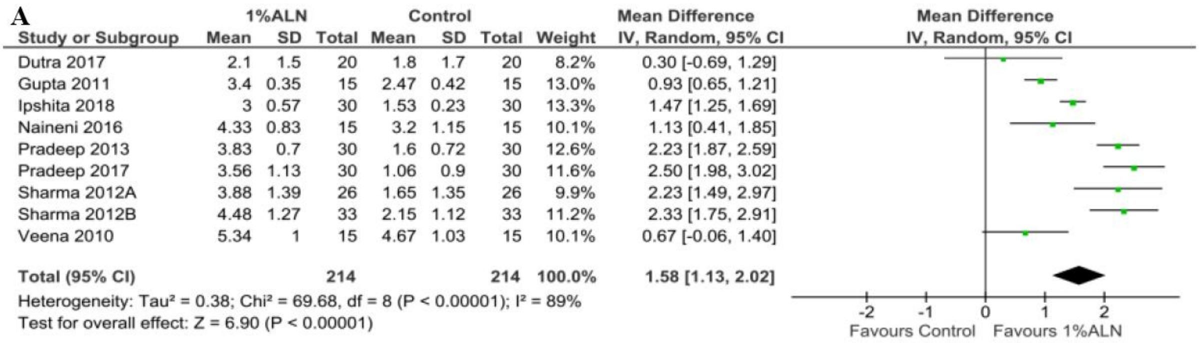

Fig. S3. Forest plot of comparison: adjunctive 1%ALN was compared with conventional periodontal treatments alone with respect to PPD reduction in patients without smoking habit or type II DM after 6 months of follow-up.

Supplemental Figure 4

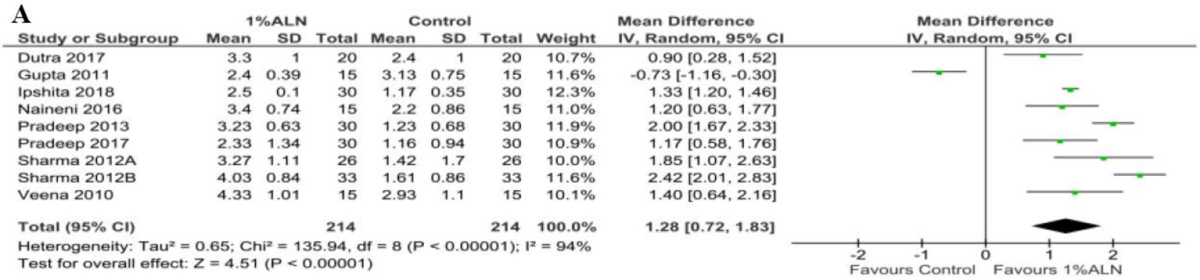

Fig. S4. Forest plot of comparison: adjunctive 1%ALN was compared with conventional periodontal treatments alone with respect to VCAL-regained in patients without smoking habit or type II DM after 6 months of follow-up.
